# Copper-catalyzed cascade reactions of α,β-unsaturated esters with keto esters

**DOI:** 10.3762/bjoc.11.23

**Published:** 2015-02-06

**Authors:** Zhengning Li, Chongnian Wang, Zengchang Li

**Affiliations:** 1 College of Environmental and Chemical Engineering, Dalian University, Dalian 116622, China

**Keywords:** aldol addition, cascade reaction, catalysis, conjugate reduction, copper, lactonization

## Abstract

A copper-catalyzed cascade reaction of α,β-unsaturated esters with keto esters is reported. It features a copper-catalyzed reductive aldolization followed by a lactonization. This method provides a facile approach to prepare γ-carboxymethyl-γ-lactones and δ-carboxymethyl-δ-lactones under mild reaction conditions.

## Introduction

Paraconic acid and its analogues, sharing the similar structure of a γ-lactone with an attached β-carboxylic acid, are widely found in natural products. Related syntheses have been actively explored due to the potential antitumor and antibiotic activities of these compounds [1–4]. The synthesis methods were mainly based on the lactonization of γ-hydroxy esters, which were obtained through either addition of substituted succinate-derived enolates to carbonyls [3–7], or direct reduction of the corresponding γ-keto esters [8]. Usually they were carried out under acidic or basic conditions, which are not suitable for some acid-sensitive or base-sensitive intermediates.

**Figure 1:**
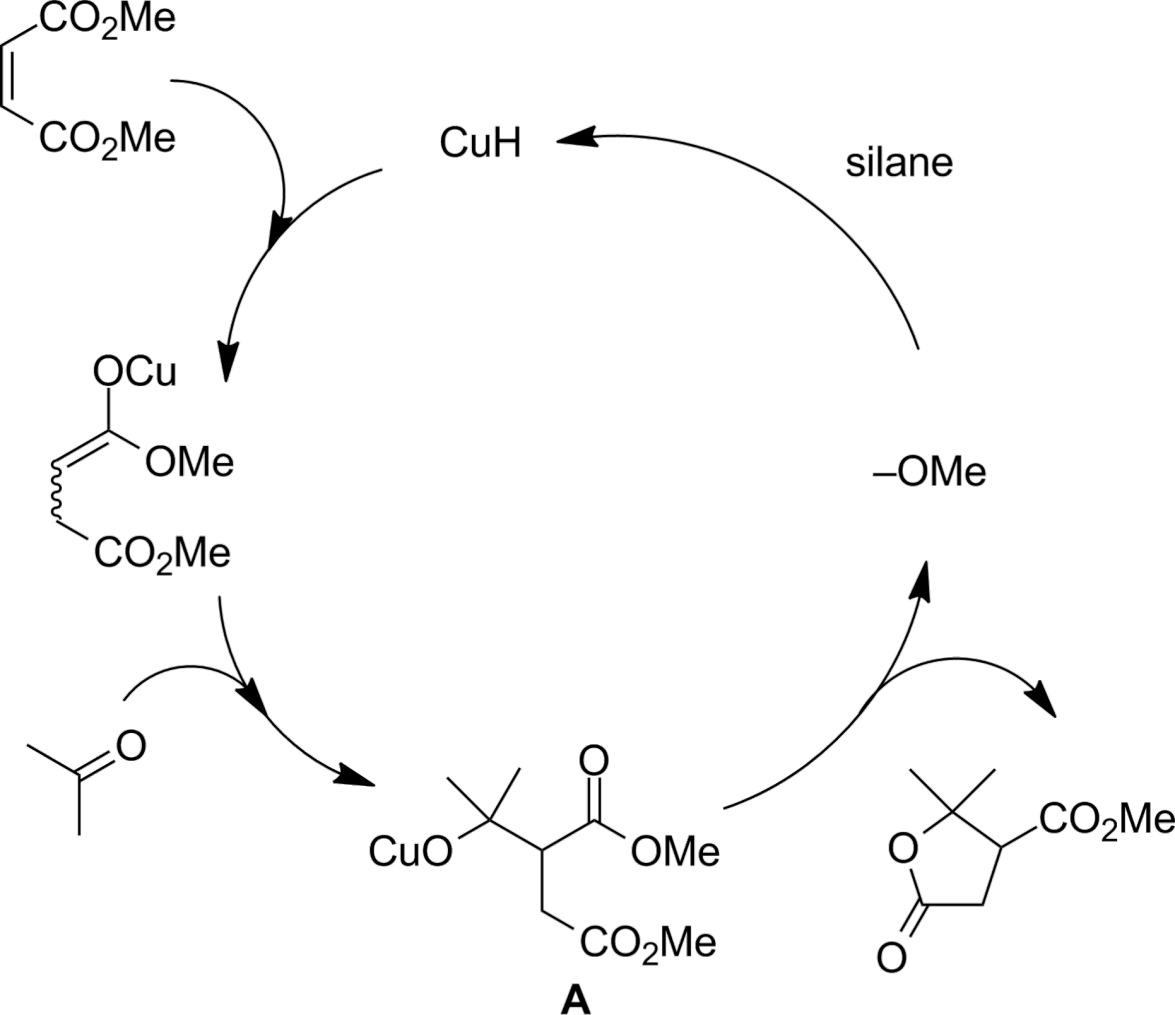
Mechanism of reductive aldol–lactonization reaction of maleate with a carbonyl and silane under copper catalysis.

Alternatively, the lactone is also formed through the reaction of a carbonyl with an enolate that can be generated from a metal-catalyzed conjugate addition of an α,β-unsaturated diester. As shown in Figure 1, it involves a conjugate reduction of the α,β-unsaturated diester with newly generated copper hydride, followed by aldol reaction to yield the key intermediate alkoxide **A**, which is subjected to further lactonization to form the lactone. Lam’s group has furnished a cobalt-catalyzed conjugate reductive aldolization–lactonization cascade reaction using diethylzinc as a reductant [9]. Floch et al. have reported a catalytic synthesis of the γ-lactones from aryl bromides, zinc, dimethyl itaconate and aldehydes/ketones [10–11], which is characteristic in its simplicity, conciseness and high yielding, however with low diastereoselectivity. As expected, a cyclic β-halo-α,β unsaturated aldehyde substrate led to the respective tricyclic lactone, the core structure of strigolactone [12]. Bertrand synthesized β-carboxylic acid-γ-lactones in high yields but with low diastereoselectivity via a dialkylzinc-mediated radical addition to the unsaturated diester followed by a sequential aldol addition and lactonization reaction [13]. To address aforementioned challenges, we have recently reported the first example of a copper-catalyzed reductive aldolization–lactonization cascade reaction of α,β-unsaturated diesters with ketones [14] and imines [15], respectively.

Although syntheses of β-carboxy-γ-lactones have been studied extensively, synthetic methods for γ-carboxymethyl-γ-lactones have been scantly explored. Taking into account the known bioactivities of the β-carboxy-γ-lactone class, its close isomer γ-carboxymethyl-γ-lactone should be of pharmaceutical interest as well. To date we have found only one example of the synthesis of the γ-carboxymethyl-γ-lactone involving the reaction of α-bromocarboxylate with zinc and a γ-oxocarboxylate [16]. To this end, we are attempting to explore its preparation and herein reporting the synthesis of γ-carboxy-γ-lactones via a copper-catalyzed cascade reaction.

**Figure 2:**
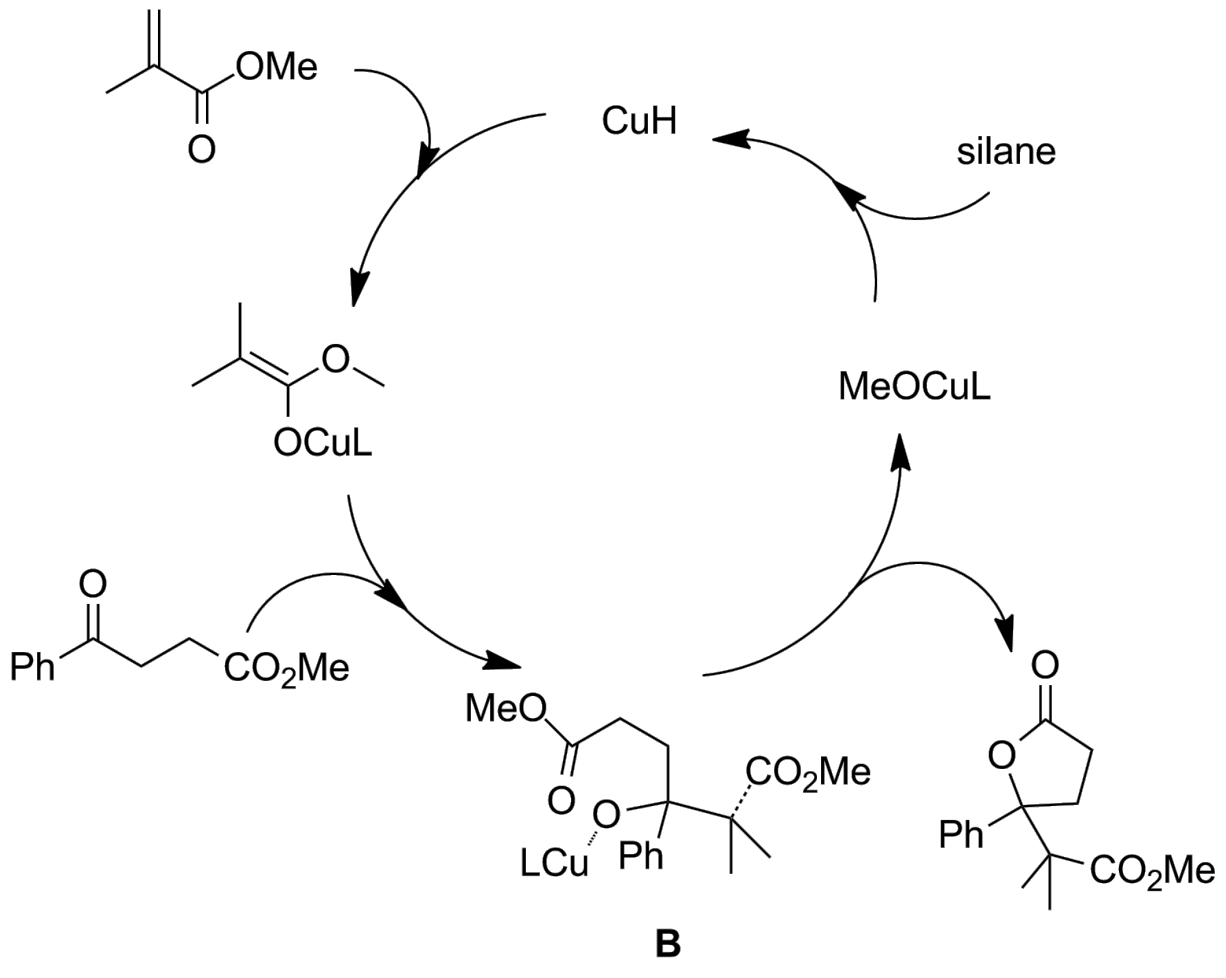
Possible copper-catalyzed reductive aldol–lactonization reaction of acrylate with a keto ester and silane.

Considering that the reported conjugate addition–aldolization–lactonization cascade reactions proceed via the key intermediate **A** in Figure 1, we envisioned that the conjugate reduction of a methacrylate and the following aldol reaction with a γ-keto ester (instead of simple ketone) would yield an intermediate **B** as described in Figure 2. Intermediate **B** possesses a γ-alkoxide unit and an ester unit as that in intermediate **A**, and therefore, it is expected to afford a γ-carboxymethyl lactone as the final product. Furthermore, change of the linker’s length is expected to yield δ-carboxymethyl-δ-lactones.

Copper-catalyzed reductive intermolecular aldolization reactions have been discussed previously [17–19]. Constantino has demonstrated that aldol reactions of enolates with ketones or aldehydes are much faster than the Claisen condensation [20]. Therefore, it is rationalized that an α,β-unsaturated monoester-derived enolate should react preferentially with the keto group in the keto ester to yield the alkoxide, which further lactonizes with an intramolecular ester group to form the lactone. It should provide a novel three-step cascade reaction, yielding the lactone with a tethered ester moiety.

## Results and Discussion

Initially, methyl methacrylate (**1a**) was employed as a copper hydride acceptor. In the presence of methyl 4-oxo-4-phenylbutanoate (**2a**), as expected it underwent the proposed cascade reaction to furnish **3aa**, a γ-lactone with a γ-exo-ester group, in 90% yield (Table 1, entry 1). Since the existing methyl methacrylate may also compete as the enolate acceptor, the high yield of **3aa** indicate that lactonization of the aldolization alkoxide **B** proceeded much faster than metathesis of **B** with a silane, which gave γ-hydroxy ester after the work-up. After lactonization, the derived methoxide reacted with a silane in a reasonable rate to recover the CuH catalyst. This is in agreement to our previously described copper-catalyzed reductive aldolization–lactonization cascade reaction of α,β-unsaturated diesters with silane and ketones [14]. Substituted phenyl keto esters gave lactones with an exo-ester group in good yields (Table 1, entries 2–5). Aliphatic keto ester **2f** also readily yielded corresponding lactone **3af** (Table 1, entry 6).

**Table 1:** Reactions of methyl 4-oxocarboxylates with methyl methacrylate.

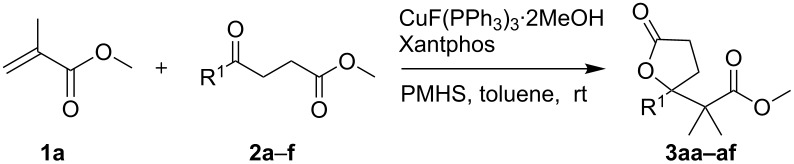			

Entry	**2** (R^1^)	Time (h)	Yield (%)

1^a^	**2a** (C_6_H_5_)	4	90
2^b^	**2b** (*o*-CH_3_OC_6_H_4_)	24	76
3^b^	**2c** (*m*-CH_3_OC_6_H_4_)	16	71
4^b^	**2d** (*p*-CH_3_OC_6_H_4_)	12	84
5^b^	**2e** (*p*-BrC_6_H_4_)	6	86
6^a^	**2f** (CH_3_)	4	85

^a^**2a** (0.25 mmol):**1**:PMHS:CuX:ligand = 1:1.5:3:1%:1%; ^b^**2**: **1**: PMHS:CuX:ligand = 1:1.5:3:2%:2%.

The use of acrylate as an α,β-unsaturated ester in the cascade reaction generated two chiral centers, and that produced a mixture of diastereomers with potential challenges in the stereoselectivities. Reaction of methyl acrylate (**1d**) with **2a** and PMHS in toluene at 25 °C gave two diastereomers which were detected by GC, however inseparable by column chromatography. Formation of the *anti*-isomer was slightly favored at 25 °C (Table 2, entry 1), and the *syn*-isomer was preferred at lower temperature, however with a slower reaction rate (Table 2, entry 3). Reaction in THF was slower compared to that in toluene (Table 2, entry 4 vs 2). In this case, the presence of biphosphorous ligands had little effect on the diastereoselectivity (Table 2, entries 5–10). The *anti*-isomer lactones were more favorable when replacing acrylate with crotonate (Table 2, entry 11).

**Table 2:** Reaction of methyl 4-oxo-4-phenylbutanoate with methyl acrylate or methyl crotonate using different ligands^a^.

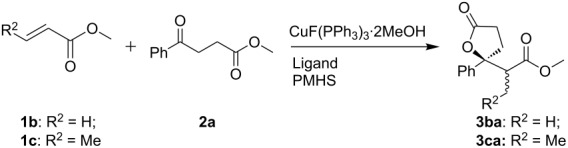						

Entry	R^2^	Ligand	*T* (°C)	Time (h)	*anti*/*syn*	Yield (%)

1^b^	H	DPEphos	25	4	58:42	94
2^b^	H	DPEphos	0	5	54:46	84
3^c^	H	DPEphos	−30	6	42:58	75
4^b,d^	H	DPEphos	0	5	58:42	53
5^b^	H	Xantphos	25	4	57:43	97
6^b^	H	DPBen	25	2	57:43	96
7^b^	H	dppm	25	4	57:43	63
8^b^	H	ddpb	25	4	56:44	91
9^b^	H	dppe	25	4	54:46	95
10^b^	H	dppp	25	4	59:41	95
11^d^	Me	Xantphos	25	8	61:39	88

^a^**2** (0.25 mmol):**1**:PMHS = 1:1.2:2, toluene as the solvent unless noted; ^b^1 mol % of CuX, 1 mol % of ligand; ^c^2 mol % of CuX, 2 mol % of ligand, ^d^THF as the solvent.

**Figure 3:**
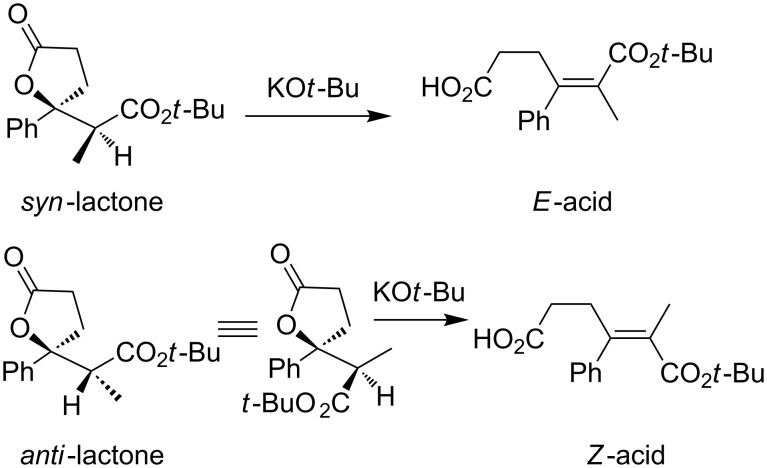
The elimination reactions of lactones.

Our previous results suggested that higher diastereoselectivity could be achieved with hindered acrylate in the copper hydride-catalyzed reductive aldolization of acetophenone [19]. Therefore, *tert-*butyl acrylate (**1d**) was used to replace methyl acrylate, and several biphosphorous ligands were screened for improving the diastereoselectivity (Table 3). Diastereomers of **3da** were separated, which were subjected to further treatment with KO*t*-Bu to form the corresponding E2 or E1_CB_ products in an effort to determine the stereochemistries assuming *trans*-elimination (Figure 3). The configurations of elimination products could be identified by NOESY. A crosspeak between phenyl and *tert*-butyl and the absence of a correlation between phenyl and the methyl group attached to the sp^2^-C are observed in the *Z*-acid, while contrary to these, absence of the phenyl–*tert*-butyl interaction and the presence of one crosspeak between phenyl and the methyl group are observed in the *E*-acid. The diastereochemistries of other lactone diastereomers were then deduced by ^1^H NMR chemical shifts of CHC*H*_3_ or OC*H*_3_. As shown in Table 3, *syn*-**3da** was favored when *tert*-butyl acrylate was used to replace the methyl acrylate (Table 3, entry 8 vs Table 2, entry 1). Higher yields were obtained in the presence of biphosphorous ligands (Table 3, entries 2–8 vs entry 1), and an effect of ligands on the diastereoselectivity of lactone was observed too. Xantphos was superior to other ligands. Overall, the diastereoselectivities were much lower than those in the reactions of *tert*-butyl acrylate with acetophenone and silane.

**Table 3:** Reactions of methyl 4-phenyl-4-oxobutanoate with *tert*-butyl acrylate^a^.

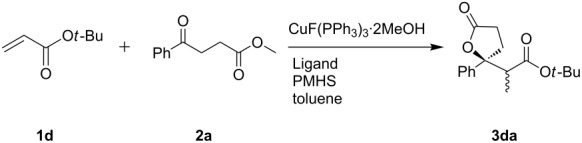					

Entry	Ligand	Time (h)	Conv. (%)	*anti/syn*	yield (%)^b^

1^c^	–	24	70	43:57	61
2	DPBen	4	98	48:52	92
3	dppp	6	93	47:53	81
4	Dppf	9	67	34:66	58
5	Dppf	12	100	25:75	97
6^d^	Xantphos	6	100	24:76	98
7	Xantphos	4	99	28:72	93
8	DPEphos	4	84	36:64	76

^a^**2a**:**1d**:PMHS:CuX:ligand =1:1.2:2:1%:1%, 25 °C unless noted, ^b^isolated yield; ^c^**2a**:**1d**:PMHS:CuX:ligand = 1:1.2:2:3%:3%; ^d^**2a**:**1d**:PMHS:CuX:ligand = 1:1.5:3:3%:3%, 0 °C.

The diastereoselectivity varied when a substituent was introduced at the phenyl group in the keto ester, but in general no significant improvement was observed (Table 4). These reactions produced the lactones in higher yields, such as in the case of bulky **3db**.

**Table 4:** Reactions of 4-oxobutanoates with *t*-butyl acrylate^a^.

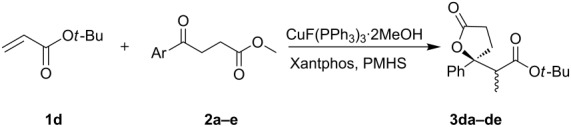				

Entry	**2** (Ar)	Time (h)	*anti/syn*	Yield (%)

1^b^	**2a** (C_6_H_5_)	4	28:72	91
2	**2b** (*o*-CH_3_OC_6_H_4_)	22	50:50	82
3	**2c** (*m*-CH_3_OC_6_H_4_)	6	29:71	91
4	**2d** (*p*-CH_3_OC_6_H_4_)	8	42:58	94
5	**2e** (*p*-BrC_6_H_4_)	6	43:57	97

^a^**2**:**1d**:PMHS:CuX:Xantphos = 1: 1.2: 2:2%:2%, 25 °C, in toluene unless otherwise noted; ^b^**2**:**1d**:PMHS:CuX:Xantphos = 1:1.2:2:1%:1%.

This cascade reaction was further extended to the reaction of 5-oxo-5-(substituted)phenylpentanoates **4** to form δ-lactones. Even though methyl methacrylate failed to give lactonized products, *tert*-butyl acrylate afforded the δ-lactones in moderate to good yields with low diastereoselectivities. The details are listed in Table 5. Formation of δ-lactone was slower than that of γ-lactone and more catalyst or longer reaction times were needed to obtain a reasonable yield of lactones (Table 5, entry 1 vs Table 4, entry 1; Table 5, entry 6 vs Table 4, entry 4).

**Table 5:** Reactions of 5-oxopentanoates with *tert*-butyl acrylate.

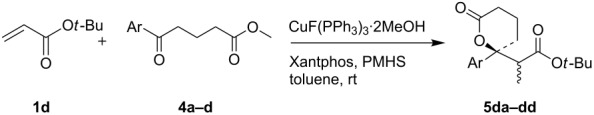					

Entry	**4** (Ar)	Time (h)	Conv. (%)	*anti*/*syn*	Yield (%)

1^a^	**4a** (C_6_H_5_)	6	91	30:70	70
2^a^	**4a** (C_6_H_5_)	8	97	26:74	90
3^a^	**4b** (*o*-CH_3_OC_6_H_4_)	19	57	62:38	31
4^b^	**4b** (*o*-CH_3_OC_6_H_4_)	23	78	62:38	60
5^b^	**4c** (*m*-CH_3_OC_6_H_4_)	6	97	19:81	90
6^b^	**4d** (*p*-CH_3_OC_6_H_4_)	23	71	33:67	55

^a^**4**:**1d**:PMHS:CuX:Xantphos = 1:1.5:3:3%:3%; ^b^**4**:**1d**:PMHS:CuX:Xantphos = 1:1.5:3:5%:5%.

## Conclusion

In conclusion, a novel copper-catalyzed three-component cascade reaction among α,β-unsaturated esters, keto esters and a silane was developed. The reaction involves conjugate addition of copper hydride, generated in situ, to α,β-unsaturated esters, followed by the aldolization with a keto ester and lactonization. It gives γ-carboxymethyl-γ-lactones and δ-carboxymethyl-δ-lactones in high yields, and provides a promising synthetic method for ester-functionalized γ-lactones and δ-lactones.

## Experimental

**A typical procedure**: Under a N_2_ atmosphere, a solution of CuF(PPh_3_)_3_·2MeOH (1.9 mg, 0.0025 mmol), and Xantphos (1.2 mg, 0.0025 mmol) in toluene (1 mL) in a Schlenk tube was stirred for 15 min. PMHS (0.040 mL) was added to the solution, and the resulting solution was stirred for 30 min. A solution of **1a** (48 mg, 0.25 mmol) and **2a** (32 mg, 0.32 mmol) in toluene (1 mL) was added slowly to the tube. The progress of the reaction was monitored by TLC. When most of **2a** was consumed, the reaction was quenched by the addition of a NH_4_F solution in methanol/H_2_O (10 mL). The mixture was filtered. The filtrate was separated, and the aqueous phase was extracted with dichloromethane (3 × 10 mL). The combined organic phase was washed with brine, dried and concentrated in vacuo. The conversion of **2a** was determined by GC. Column chromatography afforded methyl 2-methyl-2-(5-oxo-2-phenyltetrahydrofuran-2-yl)propanoate (**3aa**) (0.049 g, 90% yield) as colorless solid. mp 81–83 °C. *R*_f_ = 0.34 (EA/PE = 20:80). ^1^H NMR (400 MHz, CDCl_3_) δ 7.38–7.29 (m, 5H), 3.67 (s, 3H), 3.00–3.15 (m, 1H), 2.63–2.46 (m, 2H), 2.38–2.23 (m, 1H), 1.26 (s, 3H), 1.16 (s, 3H); ^13^C NMR (100 MHz, CDCl_3_) δ 176.22, 175.32, 140.31, 128.16, 128.03, 126.48, 91.31, 52.18, 50.05, 31.02, 28.88, 22.01, 21.27; LRMS–EI (70 ev), *m*/*z* (%): 203 (3), 161 (100), 143 (5), 133 (47), 115 (39), 105 (80), 91 (17), 77 (51), 55 (10), 51 (10); HRMS–FAB calculated for C_15_H_18_O_4_ + NH_4_^+^, 280.1543; found, 280.1550.

For those products as diastereomers, the conversion of keto esters and diastereoselectivities of lactones were determined by GC analyses.

## Supporting Information

File 1 Characterization data of the products.
