# Supplementary material for: Copper-catalyzed cascade reactions of α,β-unsaturated esters with keto esters
Source: Beilstein J Org Chem. 2015 Feb 6;11:213–8. doi: 10.3762/bjoc.11.23 (PMC4362015; doi:10.3762/bjoc.11.23)
Supplement: File 1 — Characterization data of the products. [file Beilstein_J_Org_Chem-11-213-s001.pdf]

Supporting Information

for

Copper-catalyzed cascade reactions of α,β-unsaturated esters with keto esters

Zhengning Li*, Chongnian Wang and Zengchang Li

Address: College of Environmental and Chemical Engineering, Dalian University, Dalian 116622, China

Email: Zhengning Li* - znli@dl.cn

*Corresponding author

Characterization data of the products

General

All reagents were purchased from Alfa Aesar, a Johnson Matthey company, and the solvents were bought from Sinoreagent company and used as received except those noted. Methyl 4-oxo-4-(substituted)phenylbutanoates (**2a**–**d**) and 5-oxo-5-(substituted)- phenylpentanoates **4** were prepared according to literature methods[1,2] via a reaction of 4-(substituted)phenyl magnesium bromides with methyl 4-chloro-4-oxobutanoate, and 5-oxo-5-(substituted)phenylpentanoates **4**, respectively. **2e** was prepared from an $AlCl_3$-catalyzed reaction of phenyl bromide with 4-chloro-4-oxobutanoate.[3] Structures of **2** and **4** were confirmed by their MS and IR spectra.

NMR spectra were recorded on a Varian Mercury Plus-400 NMR or a Bruker DRX500 spectrometer in $CDCl_3$ at 400 MHz or 500 MHz respectively for ^{1}H NMR, and at 100 or 125 MHz for ^{13}CNMR, respectively. LRMS spectra were recorded on a HP 6890/5973 GC-MS mass spectrometer at 70 eV. HRMS were recorded on a Micromass UPLC/Q-Tof Micro spectrometer. GC analyses were performed on a Shimadzu GC-2010 GC Chromatograph fitted with a FID detector and an SE-54 capillary column (30 m*0.32 mm*0.4 μm).

1. Babudri, F.; Fiandanese, V.; Marchese, G.; Punzi, P. *Tetrahedron,* **1996**, *52*, 13513-13520.
2. Takeuchi, T.; Matsuhashi,M.; Nakata. T. *Tetrahedron Lett.*, **2008**, *49*, 6462–6465.
3. Kooistra, F. B. ; Knol, J.; Kastenberg, F.; Popescu, L. M.; Verhees, W. H.; Kroon, J. M.; Hummelen, J. C. *Org. Lett*., **2007**, *9*, 551-554.

Characterization data

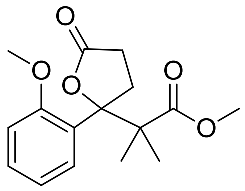

Methyl 2-(2-(2-methoxyphenyl)-5-oxotetrahydrofuran-2-yl)-2-methylpropanoate (**3ab**)

Colorless solid. m.p= 65-67 ℃. R_f = 0.31 (EA: PE = 20: 80).

^{1}H NMR (400 MHz, $CDCl_3$) δ 7.50 (d, *J* = 7.8 Hz, 1H), 7.39 – 7.24 (m, 1H), 6.96 (t, *J* = 7.6 Hz, 1H), 6.88 (d, *J* = 8.2 Hz, 1H), 3.76 (s, 3H), 3.68 (s, 3H), 3.13 (ddd, *J* = 14.3, 10.0, 7.9 Hz, 1H), 2.86 – 2.74 (m, 1H), 2.53 (ddd, *J* = 16.0, 10.1, 5.7 Hz, 1H), 2.37 (ddd, *J* = 18.2, 10.4, 7.7 Hz, 1H), 1.28 (s, 3H), 1.10 (s, 3H).

^{13}C NMR (100 MHz, $CDCl_3$) δ 176.78, 175.15, 155.47, 129.55, 129.12, 128.55, 120.39, 111.16, 91.53, 55.04, 52.02, 50.21, 29.19, 28.66, 22.23, 21.96.

LRMS (EI, 70ev), m/z (%) 191 (100), 163(81), 145 (5), 135 (22), 105(3), 91 (5), 77 (10), 55 (5).

FAB-HRMS calculated for $C_{16}H_{20}O_5$+NH_4^+310.1649, found 310.1671.

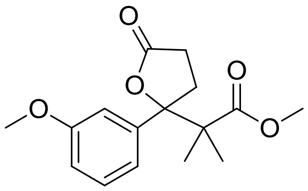

Methyl 2-(2-(3-methoxyphenyl)-5-oxotetrahydrofuran-2-yl)-2-methylpropanoate (**3ac**)

Colorless oil. R_f = 0.34 (EA: PE = 20: 80).

^{1}H NMR (400 MHz, $CDCl_3$) δ 7.29 – 7.23 (m, 1H), 6.90 (d, *J* = 7.2 Hz, 2H), 6.85 (d, *J* = 8.4 Hz, 1H), 3.80 (s, 3H), 3.68 (s, 3H), 3.08 (dd, *J* = 23.1, 10.1 Hz, 1H), 2.63 – 2.44 (m, 2H), 2.41 – 2.22 (m, 1H), 1.27 (s, 3H), 1.17 (s, 3H).

^{13}C NMR (100 MHz, $CDCl_3$) δ 176.17, 175.37, 159.35, 142.11, 129.10, 118.80, 113.25, 112.89, 91.28, 55.39, 52.19, 50.07, 31.17, 28.90, 22.13, 21.33.

LRMS (EI, 70ev), m/z (%) 292(3, M^+) 191 (100), 163(5), 145 (5), 135 (20), 107(5), 92 (3), 77 (4), 55 (2).

FAB-HRMS calculated for $C_{16}H_{20}O_5$+NH_4^+: 310.1649, found 310.1654.

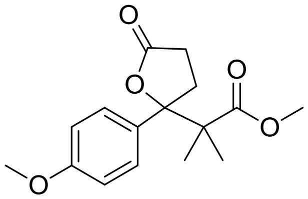

Methyl 2-(2-(4-methoxyphenyl)-5-oxotetrahydrofuran-2-yl)-2-methylpropanoate (**3ad**)

Colorless oil. R_f = 0.28 (EA: PE = 20: 80).

^{1}H NMR (400 MHz, $CDCl_3$) δ 7.26 (d, *J* = 8.1 Hz, 2H), 6.87 (d, *J* = 8.1 Hz, 2H), 3.81 (s, 3H), 3.67 (s, 3H), 3.03 (q, *J* = 10.4 Hz, 1H), 2.60 – 2.44 (m, 2H), 2.39 – 2.23 (m, 1H), 1.25 (s, 3H), 1.16 (s, 3H).

^{13}C NMR (100 MHz, $CDCl_3$) δ 176.35, 175.39, 159.28, 132.03, 127.76, 113.27, 91.23, 55.31, 52.16, 50.14, 30.92, 28.93, 21.95, 21.24.

LRMS (EI, 70ev), m/z (%) 191 (100), 164(4), 145 (3), 135 (20), 107(2), 92 (3), 77 (4), 55 (2).

FAB-HRMS calculated for $C_{16}H_{20}O_5$+NH_4^+ 310.1649, found 310.1671.

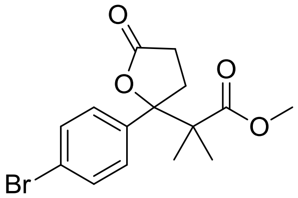

Methyl 2-(2-(4-bromophenyl)-5-oxotetrahydrofuran-2-yl)-2-methylpropanoate (**3ae**)

Colorless solid. m.p=105-106℃.R_f = 0.43 (EA: PE = 20: 80).

^{1}H NMR (400 MHz, $CDCl_3$) δ 7.48 (d, *J* = 8.7 Hz, 2H), 7.23 (d, *J* = 8.7 Hz, 2H), 3.67 (s, 3H), 3.14 – 3.04 (m, 1H), 2.58 – 2.48 (m, 2H), 2.38 – 2.26 (m, 1H), 1.26 (s, 3H), 1.15 (s, 3H).

^{13}C NMR (100 MHz, $CDCl_3$) δ 175.84, 175.14, 139.59, 131.24, 128.34, 122.49, 90.88, 52.30, 50.03, 31.01, 28.78, 22.00, 21.23.

LRMS (EI, 70ev), m/z (%) 239 (100), 211(7), 188 (22), 155 (7), 115(6), 105 (5), 76 (5), 55 (3).

FAB-HRMS calculated for $C_{15}H_{17}BrO_4$+NH_4^+: 358.0648, found 358.0657.

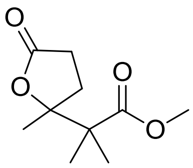

Methyl 2-methyl-2-(2-methyl-5-oxotetrahydrofuran-2-yl)propanoate (**3af**)

Colorless oil. R_f = 0.27 (EA: PE = 20: 80).

^{1}H NMR (400 MHz, $CDCl_3$) δ 3.70 (s, 3H), 2.71 – 2.48 (m, 3H), 1.95 (td, *J* = 10.4, 1.7 Hz, 1H), 1.42 (s, 3H), 1.29 (s, 6H).

^{13}C NMR (100 MHz, $CDCl_3$) δ 176.58, 175.53, 88.53, 52.19, 49.36, 30.00, 29.01, 23.47, 21.17, 21.04.

LRMS (EI, 70ev), m/z (%)185(3), 157(4), 141(8), 125(5), 113(8), 99 (100), 87 (4),71(7), 55 (7).

FAB-HRMS calculated for $C_{10}H_{16}O_4$+H^+: 201.1121, found 201.1131.

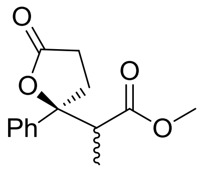

Methyl 2-(5-oxo-2-phenyltetrahydrofuran-2-yl)propanoate (**3ba**)

Diastereomeric mixture of **3ba** (*anti*:*syn* = 87:13, isomerized from a 58:42 mixture) is inseparable by column chromatography. The dr was determined by GC. R_f = 0.32 (EA: PE = 20: 80).

^1H NMR (400 MHz, $CDCl_3$) δ7.45 – 7.28 (m, 5H), 3.65 (s, 3H*0.14), 3.64 (s, 3H*0.86), 3.06 (q, *J* = 7.1 Hz, 1H*0.1), 3.03 (q, *J* = 7.1 Hz, 1H*0.9), 2.86 – 2.76 (m, 1H), 2.63 – 2.51 (m, 2H), 2.49 – 2.37 (m, 1H), 1.16 (d, *J* = 7.2 Hz, 3H*0.88, *anti*-), 1.13 (d, *J* = 7.1 Hz, 3H*0.12,,*syn*-).

major *(anti*-) ^{13}C NMR (100 MHz, $CDCl_3$) δ 176.11, 173.41, 142.42, 128.65, 128.12, 125.07, 88.41, 51.99, 50.04, 31.78, 28.74, 12.97. LRMS (EI, 70ev), m/z (%) 161(100), 133 (11), 115 (10), 105 (33), 91 (3), 77 (15), 51 (3).

Minor (*syn*-) ^{13}C NMR (100 MHz, $CDCl_3$) δ 175.88, 173.04, 140.39, 128.44, 128.39, 125.95, 89.17, 51.99, 49.57, 30.74, 28.67, 12.40.

LRMS (EI, 70ev), m/z (%) 161(100), 133 (10), 115 (9), 105 (30), 91 (3), 77 (13), 51 (3).

FAB-HRMS calculated for $C_{14}H_{16}O_4$+NH_4^+: 266.1387, found 266.1392.

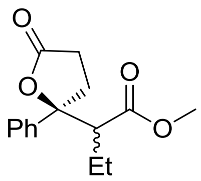

Methyl 2-(5-oxo-2-phenyltetrahydrofuran-2-yl)butanoate (**3ca**) (*anti*:*syn* = 55:45)

Colorless oil. R_f = 0.34 (EA: PE = 20: 80), one spot.

^1H NMR (400 MHz, $CDCl_3$) δ 7.42 – 7.29 (m, 5H), 3.66 (s, 3H*0.45), 3.62 (s, 3H*0.55), 3.04 – 2.93 (m, 1H*0.4), 2.86 (dd, *J* = 12.4, 3.3 Hz, 1H*0.6), 2.83 – 2.73 (m, 1H), 2.63 – 2.48 (m, 2H), 2.47 – 2.29 (m, 1H), 1.87 – 1.72 (m, 1H*0.6), 1.67 – 1.58 (m, 1H*0.4), 1.56 – 1.45 (m, 1H), 0.83 (t, *J* = 7.4 Hz, 3H*0.55), 0.82 (t, *J* = 7.4 Hz, 3H*0.45),

Major (*anti*-) ^{13}C NMR (100 MHz, $CDCl_3$) δ 176.00, 173.00, 142.46, 128.64, 128.46, 125.06, 88.50, 58.52, 51.84, 31.66, 28.73 20.99, 12.21.

LRMS (EI, 70ev), m/z (%) 161(100), 133 (6), 115 (5), 105 (18), 91 (3), 77 (8), 55 (3).

Minor (*syn*-)^{13}C NMR (100 MHz, $CDCl_3$) δ 175.79, 172.75, 140.71, 128.43, 128.13， 125.98, 89.07, 57.65, 51.84, 29.95, 28.68, 20.58, 12.18.

LRMS (EI, 70ev), m/z (%) 161(100), 133 (6), 115 (6), 105 (19), 91 (3), 77 (8), 55 (2).

FAB-HRMS calculated for $C_{15}H_{18}O_4$+NH_4^+ 280.1543, found 280.1545.

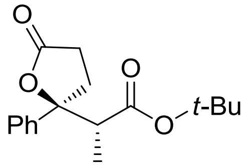

(R^*)-*tert*-Butyl 2-((S^*)-5-oxo-2-phenyltetrahydrofuran-2-yl)propanoate (*anti*-**3da**) to *Z* acid

Colorless oil. R_f = 0.63 (EA: PE = 15: 85).

^{1}H NMR (400 MHz, $CDCl_3$) δ 7.37 – 7.32 (m, 5H), 2.99 – 2.87 (m, 1H), 2.90 (q, *J* = 7.3 3Hz, 1H), 2.65 – 2.50 (m, 2H), 2.50 – 2.38 (m, 1H), 1.39 (s, 9H), 1.13 (d, *J* = 7.1 Hz, 3H).

^{13}C NMR (100 MHz, $CDCl_3$) δ 176.35, 172.34, 142.66, 128.57, 128.02, 125.30, 88.66, 81.43, 51.15, 31.51, 28.92, 28.02 ($\underline{Me_3}$C), 13.15.

LRMS (EI, 70ev), m/z (%) 217 (2, M-BuO^+), 161(100), 133 (6), 115 (14), 105 (15), 91 (1), 77 (6), 57 (9), 41 (3).

FAB-HRMS calculated for $C_{17}H_{22}O_4$+NH_4^+ 308.1856, found 308.1853.

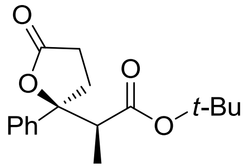

(S^*)-*tert*-Butyl 2-((S^*)-5-oxo-2-phenyltetrahydrofuran-2-yl)propanoate (*syn*-**3da**) to *E* acid

Colorless solid. m.p= 126-127 ℃. R_f = 0.57 (EA: PE = 15: 85).

^{1}H NMR (400 MHz, $CDCl_3$) δ 7.40 – 7.21 (m, 5H), 2.98 – 2.79 (m, 2H), 2.52 (dd, *J* = 14.9, 9.2 Hz, 2H), 2.32 (dd, *J* = 19.2, 9.9 Hz, 1H), 1.32 (s, 9H), 1.02 (d, *J* = 7.0 Hz, 3H).

^{13}C NMR (100 MHz, $CDCl_3$) δ 176.09, 171.83, 140.90, 128.33, 128.21, 126.01, 89.41, 81.39, 50.42, 30.82, 28.68, 28.00, 12.51.

LRMS (EI, 70ev), m/z (%) 217 (4, M-BuO^+), 161(100), 133 (10), 115 (7), 105 (16), 91 (3), 77 (10), 57 (16), 41 (4).

FAB-HRMS calculated for $C_{17}H_{22}O_4$+NH_4^+ 308.1856, found 308.1865

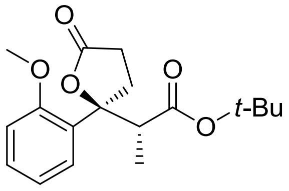

(R^*)-*tert*-Butyl 2-((S^*)-2-(2-methoxyphenyl)-5-oxotetrahydrofuran-2-yl)propanoate (*anti*-**3db**)

Colorless oil. R_f = 0.29 (EA: PE = 20: 80).

^{1}H NMR (400 MHz, $CDCl_3$) δ 7.41 (d, *J* = 7.6 Hz, 1H), 7.29 (d, *J* = 7.4 Hz, 1H), 6.95 – 6.90 (m, 2H), 3.87 (s, 3H), 3.45 (q, *J* = 7.0 Hz, 1H), 2.64 – 2.47 (m, 3H), 2.45 – 2.32 (m, 1H), 1.25 (d, *J* = 7.0 Hz, 3H), 1.08 (s, 9H).

^{13}C NMR (100 MHz, $CDCl_3$) δ 177.00, 171.76, 155.72, 130.54, 129.36, 126.71, 120.56, 111.26, 88.10, 80.18, 55.42, 47.49, 31.33, 28.40, 27.64, 11.66.

LRMS (EI, 70ev), m/z (%) 320(1, M^+), 247 (1), 191 (100), 163(51), 145 (3), 135 (14), 121 (1), 105(1), 91 (2), 77 (5), 57 (6), 41 (2), 29 (1).

FAB-HRMS calculated for $C_{18}H_{24}O_5+NH_4^+$ 338.1962, found 338.1969.

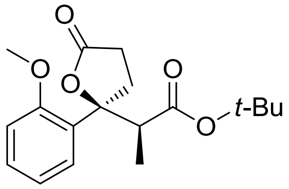

(S^*)-*tert*-Butyl 2-((S^*)-2-(2-methoxyphenyl)-5-oxotetrahydrofuran-2-yl)propanoate (*syn*-**3db**)

Colorless solid. m.p= 112-114 ℃. R_f = 0.46 (EA: PE = 20: 80).

^{1}H NMR (400 MHz, $CDCl_3$) δ 7.47 (d, *J* = 7.6 Hz, 1H), 7.30 (t, *J* = 7.8 Hz, 1H), 6.96 (t, *J* = 7.6 Hz, 1H), 6.91 (d, *J* = 8.1 Hz, 1H), 3.87 (s, 3H), 3.34 (q, *J* = 7.1 Hz, 1H), 2.97 – 2.84 (m, 1H), 2.61 (dd, *J* = 15.6, 11.2 Hz, 1H), 2.56 – 2.35 (m, 2H), 1.49 (s, 9H), 0.95 (d, *J* = 7.2 Hz, 3H).

^{13}C NMR (100 MHz, $CDCl_3$) δ 177.08, 173.02, 154.85, 131.14, 129.27, 126.68, 120.63, 111.24, 88.32, 81.23, 55.38, 47.19, 32.33, 28.80, 28.14, 13.62.

LRMS (EI, 70ev), m/z (%) 320(1, M^+), 247 (1), 191 (100), 163(48), 145 (3), 135 (13), 121 (1), 105(1), 91 (2), 77 (5), 57 (7), 41 (2), 29 (1).

FAB-HRMS calculated for $C_{18}H_{24}O_5+NH_4^+$ 338.1962, found 338.1966.

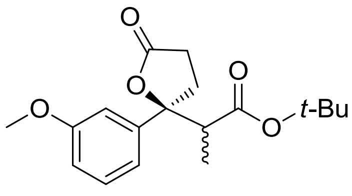

(mixture *anti*:*syn* 18:82, GC)

tert-Butyl 2-(2-(3-methoxyphenyl)-5-oxotetrahydrofuran-2-yl)propanoate (**3dc**)

Colorless oil. R_f = 0.37 (EA: PE = 20: 80), one spot.

^{1}H NMR (400 MHz, $CDCl_3$) δ 7.27 (t, J = 8.3 Hz, 1H), 7.01 – 6.81 (m, 3H), 3.81 (s, 3H), 3.00 – 2.73 (m, 2H), 2.64 – 2.33 (m, 3H), 1.41 (s, (9H*0.2), 1.40 (s, 9H*0.8), 1.12 (d, *J* = 7.2 Hz, 3H*0.20), 1.10 (d, *J* = 7.1 Hz, 3H*0.80).

anti-, ^{13}C NMR (100 MHz, $CDCl_3$) δ 176.41, 172.34, 159.75, 144.42, 129.67, 117.46, 113.20, 111.23, 88.53, 81.44, 55.42, 51.02, 31.73, 29.80, 28.87, 13.14.

syn-, ^{13}C NMR (100 MHz, $CDCl_3$) δ 176.14, 171.84, 159.59, 142.52, 129.34, 118.27, 113.49, 112.13, 89.49, 81.40, 55.44, 50.29, 30.76, 28.71, 27.99, 12.55.

LRMS (EI, 70ev), m/z (%) 320(14, M^+), 247(10), 191(100), 163(10), 145 (9), 135 (35), 107(8), 92 (6), 77 (8), 57 (17).

FAB-HRMS calculated for $C_{18}H_{24}O_5$+NH_4^+ 338.1962, found 338.1965.

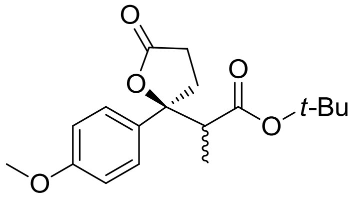

(

tert-Butyl 2-(2-(4-methoxyphenyl)-5-oxotetrahydrofuran-2-yl)propanoate (**3dd**), mixture *anti*:*syn* 42:58)**.**

Colorless oil. R_f = 0.40 (EA: PE = 20: 80), one spot.

^{1}H NMR (400 MHz, $CDCl_3$) δ 7.33 (d, *J* = 8.8 Hz, 2H*0.53), 7.29 (d, *J* = 8.8 Hz, 2H*0.47), 6.88 (d, *J* = 8.8 Hz, 2H), 3.81 (s, 3H), 2.97 –2.72 (m, 2H, 2.63 – 2.52 (m, 2H), 2.52 – 2.31 (m, 1H), 1.41 (s, 9H*0.58), 1.39 (s, 9H*0.42), 1.13 (d, *J* = 7.1 Hz, 3H*0.43), 1.07 (d, *J* = 7.1 Hz, 3H*0.57).

syn- (major) ^{13}C NMR (100 MHz, $CDCl_3$) δ 176.10, 171.91, 159.26, 132.68, 127.31, 113.61, 89.39, 81.29, 55.36, 50.51, 30.58, 28.75, 28.00, 12.49.

LRMS (EI, 70ev), m/z (%) 320(1, M^+), 191 (100), 163(3), 145 (2), 135 (12), 121 (1), 107(1), 92 (1), 77 (2), 57 (3), 41 (1).

anti- (minor) ^{13}C NMR (100 MHz, $CDCl_3$) δ 176.32, 172.31, 159.44, 134.45, 126.59, 113.80, 88.57, 81.26, 55.36, 51.17, 31.21, 28.92, 27.97, 13.09.

LRMS (EI, 70ev), m/z (%) 320(1, M^+), 191 (100), 163(3), 145 (1), 135 (13), 121 (1), 105(1), 91 (2), 77 (2), 57 (4), 41 (1).

FAB-HRMS calculated for $C_{18}H_{24}O_5$+NH_4^+ 338.1962, found 338.1992.

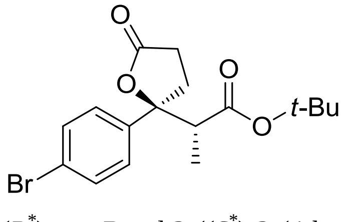

(R^*)-*tert*-Butyl 2-((S^*)-2-(4-bromophenyl)-5-oxotetrahydrofuran-2-yl)propanoate (*anti*-**3de**)

Colorless oil. R_f = 0.43 (EA: PE = 20: 80).

Data obtained from a 75/25 mixture.

^{1}H NMR (400 MHz, $CDCl_3$) δ 7.50 (d, *J* = 8.5 Hz, 2H), 7.26 (d, *J* = 8.6 Hz, 2H), 2.96 – 2.77 (m, 2H), 2.66 – 2.34 (m, 3H), 1.40 (s, 9H), 1.13 (d, *J* = 7.1 Hz, 3H).

^{13}C NMR (100 MHz, $CDCl_3$) δ 175.96, 172.10, 141.79, 131.71, 127.17, 122.17, 88.19, 81.62, 50.96, 31.46, 29.82, 28.02, 13.13.

LRMS (EI, 70ev), m/z (%) 241 (100), 211(4), 183 (12), 157(4), 129(2), 115 (4), 104(5), 57 (15).

FAB-HRMS calculated for $C_{17}H_{21}BrO_4$+NH_4^+ 386.0961, found 388.0959.

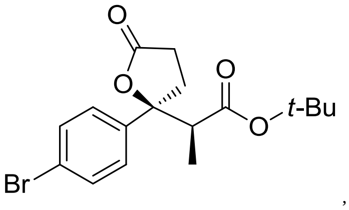

(S^*)-*tert*-Butyl 2-((S^*)-2-(4-bromophenyl)-5-oxotetrahydrofuran-2-yl)propanoate (*syn*-**3de**)

Colorless solid. m.p= 121-123 ℃. R_f = 0.41 (EA: PE = 20: 80).

^{1}H NMR (400 MHz, $CDCl_3$) δ 7.50 (d, *J* = 8.4 Hz, 2H), 7.30 (d, *J* = 8.5 Hz, 2H), 2.99 – 2.85 (m, 2H), 2.65 – 2.49 (m, 2H), 2.44 – 2.31 (m, 1H), 1.40 (s, 9H), 1.08 (d, *J* = 7.1 Hz, 3H).

^{13}C NMR (100 MHz, $CDCl_3$) δ 175.75, 171.54, 139.80, 131.42, 127.85, 122.41, 88.91, 81.60, 50.21, 31.01, 28.55, 27.97, 12.48.

LRMS (EI, 70ev), m/z (%) 239 (100), 211(4), 183 (12), 157(4), 129(4), 115 (4), 104(4), 57 (15).

FAB-HRMS calculated for $C_{17}H_{21}BrO_4$+NH_4^+ 386.0961, found 388.0951.

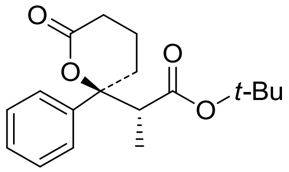

(R^*)-*tert*-Butyl 2-((S^*)-6-oxo-2-phenyltetrahydro-2*H*-pyran-2-yl)propanoate (*anti*-**5da**)

Colorless oil. R_f = 0.46 (EA: PE = 20: 80).

^{1}H NMR (400 MHz, $CDCl_3$) δ 7.40 – 7.29 (m, 5H), 2.84 (q, *J* = 7.0 Hz, 1H), 2.58 – 2.54 (m, 1H), 2.42 – 2.38(m, 2H), 2.19 (td, *J* = 13.8, 4.1 Hz, 1H), 1.80 – 1.72 (m, 1H), 1.60 – 1.51 (m, 1H), 1.35 (s, 9H), 1.14 (d, *J* = 7.1 Hz, 3H).

^{13}C NMR (100 MHz, $CDCl_3$) δ 172.29, 171.15, 141.84, 128.68, 127.84, 125.85, 87.11, 80.97, 52.43, 29.57, 28.81, 28.01 ($\underline{Me_3}$C), 16.25, 12.57.

LRMS (EI, 70ev), m/z (%) 231(3, M-BuO$^+$), 175 (100), 147(52), 129(5), 105(35), 91 (5), 77 (11), 57 (15).

FAB-HRMS calculated for $C_{18}H_{24}O_4$+NH_4^+ 322.2013, found 322.2026.

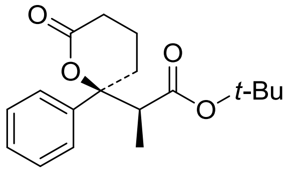

(S^*)-*tert*-Butyl 2-((S^*)-6-oxo-2-phenyltetrahydro-2*H*-pyran-2-yl)propanoate (*syn*-**5ca**)

Colorless oil. R_f = 0.40 (EA: PE = 20: 80).

^{1}H NMR (400 MHz, $CDCl_3$) δ 7.41 – 7.29 (m, 5H), 2.88 (q, *J* = 7.1 Hz, 1H), 2.49 – 2.33 (m, 4H), 1.81 – 1.68 (m, 1H), 1.63 – 1.52 (m, 1H), 1.39 (s, 9H), 1.08 (d, *J* = 7.1 Hz, 3H).

^{13}C NMR (100 MHz, $CDCl_3$) δ 172.18, 170.84, 140.88, 128.50, 127.97, 126.32, 87.87, 81.04, 51.80, 29.30, 28.04 ($\underline{Me_3}$C), 27.59, 16.41, 12.39.

LRMS (EI, 70ev), m/z (%) 231(2, M-BuO^+), 175 (100), 147(36), 129(4), 105(23), 91 (3), 77 (7), 57 (11).

FAB-HRMS calculated for $C_{18}H_{24}O_4+NH_4^+$322.2013, found 322.2018.

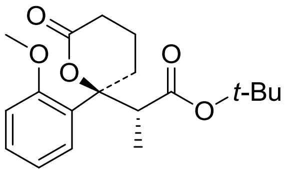

(R^*)-*tert*-Butyl 2-((S^*)-2-(2-methoxyphenyl)-6-oxotetrahydro-2*H*-pyran-2-yl)propanoate (*anti*-**5db**)

Colorless oil. R_f = 0.31 (EA: PE = 20: 80).

^{1}H NMR (400 MHz, $CDCl_3$) δ 7.35 – 7.19 (m, 2H), 6.96 – 6.90 (m, 2H), 3.85 (s, 3H), 3.42 (q, *J* = 6.8 Hz, 1H), 2.73 (d, *J* = 13.6 Hz, 1H), 2.45– 2.35 (m, 2H), 1.97 (t, *J* = 13.4 Hz, 1H), 1.67 (s, br, 1H), 1.41 – 1.32 (m, 1H), 1.28 (d, *J* = 6.8 Hz, 3H), 1.08 (s, 9H).

^{13}C NMR (100 MHz, $CDCl_3$) δ 171.80, 171.68, 155.63, 130.07, 129.27, 128.48, 120.80, 111.54, 87.63, 79.91, 55.45, 47.75, 29.65, 28.26, 27.66, 16.61, 11.98.

LRMS (EI, 70ev), m/z (%) 334(1, M^+), 261(1), 205 (100), 177(14), 165(56), 135(26), 105(2), 91 (3), 77 (5), 57 (6).

FAB-HRMS calculated for $C_{19}H_{26}O_5+NH_4^+$ 352.2118, found 352.2123.

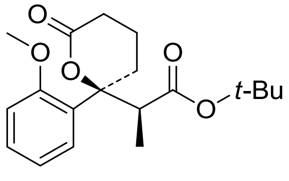

(S^*)-*tert*-Butyl 2-((S^*)-2-(2-methoxyphenyl)-6-oxotetrahydro-2*H*-pyran-2-yl)propanoate (*syn*-**5db**)Colorless solid. m.p= 127-129 ℃. R_f = 0.44 (EA: PE = 20: 80).

^{1}H NMR (400 MHz, $CDCl_3$) δ 7.34 – 7.29 (m, 2H), 6.97 (t, *J* = 7.0 Hz, 1H), 6.91 (d, *J* = 7.8 Hz, 1H), 3.85 (s, 3H), 3.44 (d, *J* = 6.8 Hz, 1H), 2.80 (d, *J* = 13.7 Hz, 1H), 2.51 – 2.35 (m, 2H), 2.25 – 2.19 (m, 1H), 1.66 (s, br, 1H), 1.47 (s, 9H), 1.39 – 1.32 (m, 1H), 0.94 (d, *J* = 6.6 Hz, 3H).
^{13}C NMR (100 MHz, $CDCl_3$) δ 172.81, 171.43, 164.09, 154.88, 129.59, 129.36, 128.76, 120.89, 111.52, 87.89, 80.77, 55.41, 47.54, 30.35, 29.17, 28.22, 17.07, 12.64.
LRMS (EI, 70ev), m/z (%) 334(1, M^+), 261(1), 205 (100), 177(14), 165(55), 135(26), 105(3), 91 (3), 77 (5), 57 (6).
FAB-HRMS calculated for $C_{19}H_{26}O_5$+NH_4^+ 352.2118, found 352.2117.

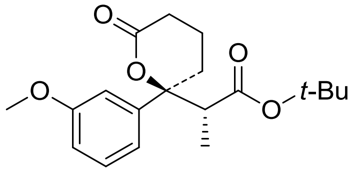

(S^*)-*tert*-Butyl 2-((S^*)-2-(3-methoxyphenyl)-6-oxotetrahydro-2*H*-pyran-2-yl)propanoate (*anti*-**5dc**)
Colorless oil. R_f = 0.46 (EA: PE = 20: 80).
^{1}H NMR (400 MHz, $CDCl_3$) δ 7.28 (t, *J* = 7.6 Hz, 1H), 6.90 (d, *J* =.7.6 Hz, 1H), 6.89 (s, 1H), 6.83 (d, *J* = 7.8 Hz, 1H), 3.81 (s, 3H), 2.82 (q, *J* = 6.8 Hz, 1H), 2.53- – 2.50 (m, 1H), 2.44 – 2.39 (m, 2H), 2.20 – 2.13 (m, 1H), 1.76 – 1.73 (m, 1H), 1.61 – 1.51 (m, 1H), 1.37 (s, 9H), 1.14 (d, *J* = 6.9 Hz, 3H).
^{13}C NMR (100 MHz, $CDCl_3$) δ 172.28, 171.09, 159.92, 143.63, 129.76, 118.11, 112.95, 112.06, 87.03, 81.00, 55.41, 52.33, 29.62, 29.08, 28.04, 16.29, 12.55.
LRMS (EI, 70ev), m/z (%) 334(6, M^+), 261(3), 205 (100), 177(44), 135(24), 107(4), 92 (2), 77 (3), 57 (8).
FAB-HRMS calculated for $C_{19}H_{26}O_5$+NH_4^+ 352.2118, found 352.2126.

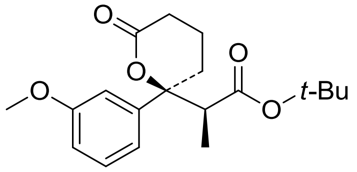

(S^*)-*tert*-Butyl 2-((S^*)-2-(3-methoxyphenyl)-6-oxotetrahydro-2*H*-pyran-2-yl)propanoate (*syn*-**5dc**)
Colorless oil. R_f = 0.40 (EA: PE = 20: 80).
^{1}H NMR (400 MHz, $CDCl_3$) δ 7.28 (t, *J* = 8.1 Hz, 1H), 6.95 (s, 1H), 6.90 (d, *J* = 7.9 Hz, 1H), 6.85 (d, *J* = 7.7 Hz, 1H), 3.81 (s, 3H), 2.87 (q, *J* = 6.9 Hz, 1H), 2.51 – 2.34 (m, 4H), 1.83 – 1.75 (m, 1H), 1.68 – 1.55 (m, 1H), 1.40 (s, 9H), 1.09 (d, *J* = 7.0 Hz, 3H).
^{13}C NMR (100 MHz, $CDCl_3$) δ 172.17, 170.82, 159.82, 142.62, 129.48, 118.59, 113.09, 112.58, 87.82, 81.03, 55.44, 51.71, 29.33, 28.04 ($\underline{Me}_3C$), 27.54, 16.44, 12.44.
LRMS (EI, 70ev), m/z (%) 334(6, M^+), 261(5), 205 (100), 177(45), 135(29), 107(4), 92 (2), 77 (4), 57 (8).

FAB-HRMS calculated for $C_{19}H_{26}O_5+NH_4^+$ 352.2118, found 352.2120.

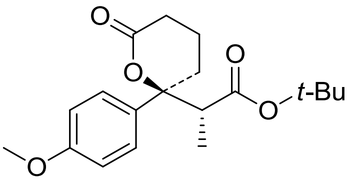

Mixture of *tert*-butyl 2-(2-(4-methoxyphenyl)-6-oxotetrahydro-2*H*-pyran-2-yl)propanoate (**5dd**).

anti-**5dd**:*syn*-**5dd** = 59:41 (GC)**.**

Colorless oil. R_f = 0.38 (EA: PE = 20: 80), one spot.

^{1}H NMR (400 MHz, $CDCl_3$) δ 7.31 (d, *J* = 8.4 Hz, 2H*048), 7.26 (d, *J* = 8.4 Hz, 2H*0.52), 6.89 (d, *J* = 7.5 Hz, 2H), 3.81 (s, 3H), 2.91 – 2.76 (m, 1H), 2.54 (dt, *J* = 15.4, 3.3 Hz, 1H*0.59), 2.47 – 2.27 (m, 3H), 2.15 (td, *J* = 13.5, 4.1 Hz, 1H*0.6), 1.90 – 1.71 (m, 1H), 1.67 – 1.51 (m, 1H), 1.41 (s, 9H*0.43), 1.35 (s, 9H*0.57), 1.14 (d, *J* = 7.1 Hz, 3H*0.58), 1.05 (d, *J* = 7.1 Hz, 3H*0.42).

anti- (major)^{13}C NMR (100 MHz, $CDCl_3$) δ 172.36, 170.96, 159.11, 133.76, 127.13, 113.94, 86.97, 80.89, 55.38, 52.61, 29.50, 28.60, 28.02, 16.23, 12.58.

LRMS (EI, 70ev), m/z (%) 334(1, M^+), 261(1), 205 (100), 177(33), 135(19), 121(2), 107(2), 92 (1), 77 (3), 57 (5).

syn-- (minor)**,** ^{13}C NMR (100 MHz, $CDCl_3$) δ172.32, 171.29, 159.21, 132.73, 127.61, 113.77, 87.76, 80.97, 55.38, 51.95, 29.24, 28.05, 27.34, 16.42, 12.38.

LRMS (EI, 70ev), m/z (%) 334(1, M^+), 261(1), 205 (100), 177(30), 135(19), 121(3), 107(1), 92 (2), 77 (3), 57 (5).

FAB-HRMS calculated for $C_{19}H_{26}O_5+NH_4^+$ 352.2118, found 352.2133.

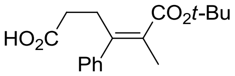

^{1}H NMR (400 MHz, $CDCl_3$) δ 11.23 (s, br, 1H), 7.35 (t, *J* = 7.4 Hz, 2H), 7.31 – 7.25 (m, 1H), 7.11 (d, *J* = 7.5 Hz, 2H), 2.92 – 2.82 (m, 2H), 2.44 – 2.35 (m, 2H), 1.69 (s, 3H), 1.54 (s, 9H).

^{13}C NMR (101 MHz, $CDCl_3$) δ 178.91, 169.62, 144.38, 140.75, 128.56, 128.46, 128.15, 127.47, 81.43, 32.70, 31.31, 28.30, 17.69.

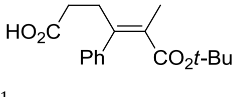

^{1}H NMR (400 MHz, $CDCl_3$) δ 10.40 (s, br, 1H), 7.31 – 7.26 (m, 3H), 7.11 (d, *J* = 6.6 Hz, 2H), 2.79 – 2.70 (m, 2H), 2.35 – 2.24 (m, 2H), 2.02 (s, 3H), 1.09 (s, 9H).

^{13}C NMR (101 MHz, $CDCl_3$) δ 178.94, 169.82, 142.74, 141.75, 129.24, 128.23, 128.08, 127.25, 31.77, 29.70, 27.55, 16.05.
